# Supplementary material for: Recent Molecular Epidemiology of Echovirus 11 Throughout North and West Africa Resulted in the First Identification of a Recombinant Strain from an Acute Flaccid Paralysis Case in West Africa
Source: Viruses. 2024 Nov 13;16(11):1772. doi: 10.3390/v16111772 (PMC11599147; doi:10.3390/v16111772)
Supplement: Supplementary file 1 [file viruses-16-01772-s001.zip › viruses-3240983-supplementary.pdf]

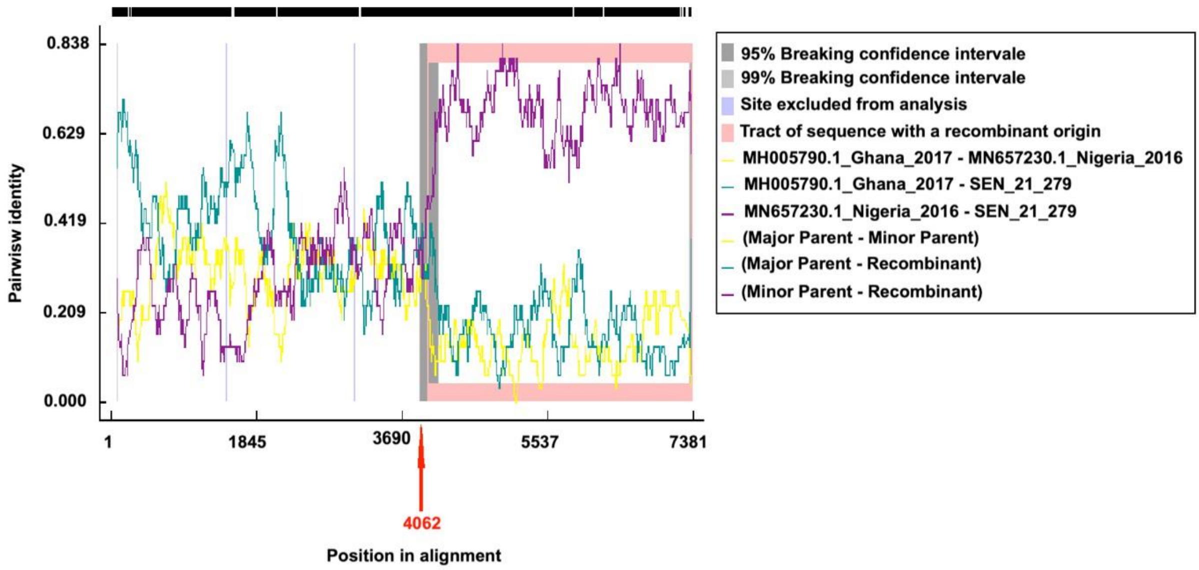

Figure S1. Recombination analysis of whole-genome sequence of the 21-279-SEN-2021 strain using RDP5. The X axis shows the nucleotide position number and Y axis shows the pairwise identity between E11 colored by green, purple and yellow line. The long black lines above the plot equivalent to the high number of nucleotide variation among sequences and the white spaces, identify regions where the polymorphism are absent. A recombination event starting at position 4062 with MN657230.1 being the minor parent.
